# Future anxiety and psychological wellbeing among sport sciences students: the mediating role of mental toughness and the moderating roles of income level and gender

**DOI:** 10.3389/fpsyg.2026.1909115

**Published:** 2026-09-23

**Authors:** Çetin Tan, Önder Şanlı, Mert Özgüner, Zeynep Özgüner, Abdullah Bingölbalı, Esin Çağla Çağlar, İrem Türkmen, Yunus Emre Büyükbasmacı, İzzet Karakulak, İsmail Belli, Serdar Koç

**Affiliations:** 1 Department of Physical Education and Sports Teaching, Faculty of Sports Sciences, Firat University, Elazig, Türkiye; 2 Department of Knowledge Management, Besni Ali Erdemoğlu Vocational School, Adıyaman University, Adıyaman, Türkiye; 3 Department of Management and Organization, Besni Ali Erdemoğlu Vocational School, Adıyaman University, Adıyaman, Türkiye; 4 Department of Business Administration, Faculty of Economics, Administrative and Social Sciences, Hasan Kalyoncu University, Gaziantep, Türkiye; 5 Department of Recreation, Faculty of Sports Sciences, Firat University, Elazig, Türkiye; 6 Department of Recreation, Faculty of Sports Sciences, Hitit University, Corum, Türkiye; 7 Department of Sport Management, Faculty of Sports Sciences, Hitit University, Corum, Türkiye; 8 Department of Recreation Management, Faculty of Applied Sciences, Karamanoglu Mehmetbey University, Karaman, Türkiye; 9 Department of Sports Management, Faculty of Sports Sciences, Mardin Artuklu University, Mardin, Türkiye; 10 Department of Exercise and Sport Sciences, Faculty of Sports Sciences, Halic University, Istanbul, Türkiye; 11 Ministry of Education, Muş, Türkiye

**Keywords:** future anxiety, mediation, mental toughness, moderation, psychological wellbeing, sport sciences students

## Abstract

**Background:**

Investigating the mechanisms of future anxiety requires a sample exposed to both high career uncertainty and intense performance pressures. Sport sciences students represent a uniquely suited and critical population for this purpose, as their educational context simultaneously demands rigorous physical performance, highly competitive labor conditions, and the management of career-related ambiguities. Therefore, this study focuses on this specific sample to explore how future anxiety is associated with psychological wellbeing and whether mental toughness may serve as a protective mechanism.

**Methods:**

This cross-sectional observational study examined 423 sport sciences faculty students. Participants completed the Future Anxiety Scale, Mental Toughness Inventory, and Psychological WellBeing Scale. Descriptive statistics, correlation analyses, regression analyses, structural equation modeling, mediation analysis, and moderation analyses were conducted to examine the relationships among future anxiety, mental toughness, psychological wellbeing, gender, and income level.

**Results:**

Future anxiety was negatively associated with psychological wellbeing (*r* = –0.378, *p* < 0.001) and mental toughness (*r* = –0.340, *p* < 0.001). Mental toughness was strongly and positively associated with psychological wellbeing (*r* = 0.770, *p* < 0.001) and partially mediated the relationship between future anxiety and psychological wellbeing [indirect effect: β = –0.247, 95% CI (–0.186, –0.099)]. Income level significantly moderated the association between future anxiety and psychological wellbeing (interaction β = 0.190, *p* < 0.001), indicating that the negative effect of future anxiety weakened as income level increased; however, given the highly imbalanced income groups, this finding should be interpreted as exploratory. Multi-group analyses suggested gender differences in some structural path coefficients (Δχ^2^ = 18.924, *p* < 0.001); however, the indirect mediation mechanism remained invariant across male and female students (Δχ^2^ = 0.000).

**Conclusion:**

Analyses of the measurement sub-dimensions indicate that the “hopelessness about the future” sub-dimension of future anxiety shows a stronger association with the outcomes than “fear of the future.” Hopelessness was associated with the largest negative coefficients for both mental toughness and overall psychological wellbeing. These findings suggest that mental toughness may be a particularly relevant mediator in relation to future-related hopelessness. Pending confirmation through longitudinal or experimental designs, interventions addressing hopelessness may be a useful focus for supporting mental toughness and wellbeing among sport sciences students.

## 1 Introduction

The university period is traditionally viewed as a time of self-discovery and academic development; however, for sport sciences students, it is simultaneously an arena of intense physical demands and profound psychological pressure. These students stand at a unique crossroads where the pursuit of peak athletic performance collides with post-graduation uncertainties and increasing competition in the labor market. With youth unemployment rates reaching alarming levels (Turkish Statistical Institute [TURKSTAT], 2020), the transition from university to professional life is fraught with ambiguity. This intense, future-oriented uncertainty breeds a specific form of apprehension known as future anxiety, conceptualized as a persistent neurotic anxiety regarding adverse outcomes (Zaleski, 1996; Geylani and Çiriş Yıldız, 2022; Kula and Saraç, 2016). Previous studies consistently highlight that unemployment and career anxiety act as major sources of psychological pressure during university education (Aytaç and Keser, 2002; Çakmak and Hevedanlı, 2005). For sport sciences students, who must continuously push their physical limits while managing these concrete economic concerns, future anxiety directly threatens their psychological wellbeing—their capacity to manage existential challenges and maintain adaptive functioning (Ryff, 1989; Telef, 2013).

To counteract the detrimental effects of stress and anxiety on psychological health, individuals rely on specific psychological resources (Gerber et al., 2018; Hu et al., 2015). In the demanding context of sport sciences, “mental toughness” emerges as a particularly vital construct. Defined as the capacity to maintain focus, confidence, and goal-directed persistence under pressure, mental toughness allows individuals to recover effectively from setbacks throughout their preparation and performance processes (Gucciardi et al., 2015; Jones et al., 2007; Cowden, 2017). The literature suggests that resilient individuals with high mental toughness are better equipped to appraise stressful situations as manageable challenges rather than debilitating threats. While previous research independently links future anxiety to decreased psychological wellbeing, and mental toughness to adaptive coping mechanisms, the interconnected dynamics of these three phenomena remain an underexplored domain, especially within populations uniquely conditioned for mental and physical endurance.

This study distinguishes itself by integrating these constructs alongside demographic realities—such as the influence of gender on cognitive and motivational processes (Pajares, 2003) and socioeconomic status on future expectations (İleri, 2019)—into a unified theoretical framework. Moving beyond the simple cause-and-effect relationships typically examined in the literature, the present study proposes a novel mechanism: future anxiety is negatively associated with psychological wellbeing, which may be partially explained by lower levels of student’s mental toughness. By examining this mediation pathway, along with the protective buffering role of income level and gender dynamics, this research offers a comprehensive model of student vulnerability and resilience. Therefore, the primary aim of this study is to investigate whether mental toughness mediates the relationship between future anxiety and psychological wellbeing among sport sciences students, and to explore how demographic variables moderate these effects. The findings offer substantial original value by demonstrating that “hopelessness about the future” is a particularly impactful element of anxiety, and that mental toughness serves as an important psychological resource, ultimately contributing to targeted, evidence-based interventions for young adults facing highly competitive professional arenas.

## 2 Materials and methods

### 2.1 Participants

This cross-sectional observational study was conducted with 423 sport sciences faculty students recruited through convenience sampling. Of the participants, 57.2% were female (*n* = 242) and 42.8% were male (*n* = 181). The sample was predominantly composed of young adults, with 48.0% falling in the 18–21 age range and 36.9% in the 22–24 range. In terms of family income, the large majority of participants (81.1%, *n* = 343) reported a middle-income household background (Table 1).

**TABLE 1:** Demographic characteristics of the participants.

Variable	Category	N*	%
Gender	Male	181	42.8
	Female	242	57.2
Age group	18–21	203	48.0
	22–24	156	36.9
	25–27	21	5.0
	28 and above	43	10.2
Family income	Low (income < expenses)	36	8.5
	Middle (income = expenses)	343	81.1
	High (income > expenses)	44	10.4
Region of residence	Mediterranean	15	3.5
	Eastern Anatolia	15	3.5
	Aegean	14	3.3
	Southeastern Anatolia	7	1.7
	Central Anatolia	10	2.4
	Black Sea	331	78.3
	Marmara	31	7.3
Number of siblings	1	40	9.5
	2	139	32.9
	3	157	37.1
	4	52	12.3
	5 and higher	35	8.3
Antidepressant use	Yes	27	6.4
	No	396	93.6
Psychological treatment history	Yes	45	10.6
	No	378	89.4

*N = 423.

Additionally, as part of the demographic information form, participants were asked single-item yes/no questions regarding their current antidepressant use and any history of psychological treatment, which were utilized solely for exploratory profile analyses.

### 2.2 Measurement instruments

#### 2.2.1 Future anxiety scale

Future anxiety was assessed using the Future Anxiety Scale, developed by Geylani and Çiriş Yıldız (2022), to measure university students’ levels of future-oriented apprehension, fear, and hopelessness. The scale consists of 19 items rated on a 5-point Likert-type scale (1 = never, 5 = always) and yields two sub-dimensions: “Fear of the Future” (13 items) and “Hopelessness about the Future” (6 items, which are reverse-scored). In the present study, construct validity was examined via confirmatory factor analysis (CFA), which indicated an acceptable fit for the two-factor structure (CFI = 0.881, RMSEA = 0.097, SRMR = 0.059). Convergent validity was supported by Average Variance Extracted (AVE) and Composite Reliability (CR) values (0.563 and 0.943 for the Fear sub-dimension; 0.503 and 0.855 for the Hopelessness sub-dimension, respectively). Internal consistency was strong, with an overall Cronbach’s alpha of 0.926 and a McDonald’s omega of 0.943 (Table 2).

**TABLE 2:** Exploratory and confirmatory factor analysis results for the psychological wellbeing scale.

Items	EFA factor loading	CFA factor loading
1. I lead a purposeful and meaningful life.	0.707	0.707
2. My social relationships are supportive and rewarding	0.799	0.799
3. I am engaged and interested in my daily activities	0.634	0.634
4. I actively contribute to the happiness and wellbeing of others	0.693	0.693
5. I am competent and capable in the activities that are important to me	0.700	0.700
6. I am a good person and live a good life	0.823	0.823
7. I am optimistic about my future	0.682	0.682
8. People respect me	0.774	0.774
Model fit statistics		
Eigenvalue	4.252	
Variance explained (%)	53.1	
KMO/Bartlett’s test	0.893/Chi-square = 1781.6, *p* < 0.001	
χ^2^/df	84.69/20	
CFI/TLI	0.913/0.978	
RMSEA [%90 CI]	0.035 [0.017–0.054]	
SRMR	0.050	
AVE	0.531	0.531
CR	0.900	0.900
Cronbach’s Alpha/McDonald Omega	0.897/0.900	

EFA, Exploratory Factor Analysis (oblimin rotation, ML estimator); CFA, Confirmatory Factor Analysis (MLR estimator); AVE, Average Variance Extracted; CR, Composite Reliability. *p < 0.05. **p < 0.01. ***p < 0.001. EFA and CFA were conducted on the same sample (*N* = 423) due to sample size constraints; results should therefore be interpreted with the caution noted in the Limitations section.

#### 2.2.2 Mental toughness inventory

Mental toughness was measured using the Mental Toughness Inventory, originally developed by Gucciardi et al. (2015) and adapted into Turkish by Etlioğlu and Yılmaz (2025). This scale is designed to assess an individual’s capacity to maintain focus, self-belief, goal-directed persistence, and emotional control under pressure. It comprises 8 items rated on a 7-point Likert-type scale (1 = strongly disagree, 7 = strongly agree) and features a unidimensional (single-factor) structure. In the current sample, CFA results demonstrated adequate model fit (CFI = 0.903, TLI = 0.864, RMSEA = 0.060, SRMR = 0.053). Convergent validity was confirmed with an AVE of 0.595 and a CR of 0.921. The scale exhibited excellent internal consistency, yielding a Cronbach’s alpha of 0.918 and a McDonald’s omega of 0.921 (Table 3).

**TABLE 3:** Exploratory and confirmatory factor analysis results for the mental toughness inventory.

Items	EFA factor loading	CFA factor loading
1. I believe in my ability to achieve my goals	0.780	0.780
2. I am able to regulate my focus when performing a task	0.667	0.667
3. I am able to use my emotions to perform the way I want to	0.767	0.767
4. I strive for continued success	0.799	0.799
5. I execute whatever knowledge is required to achieve my goals	0.811	0.811
6. I consistently overcome adversity	0.748	0.748
7. I am able to execute appropriate skills and knowledge when challenged	0.864	0.864
8. I find a positive in most situations	0.719	0.719
Model fit statistics		
Eigenvalue	4.761	
Variance explained (%)	59.5	
KMO/Bartlett’s test	0.903/Chi-square = 2244.4, *p* < 0.001	
χ^2^/df	97.57/20	
CFI/TLI	0.903/0.864	
RMSEA [%90 CI]	0.060 [0.042–0.079]	
SRMR	0.053	
AVE	0.595	0.595
CR	0.921	0.921
Cronbach’s Alpha/McDonald Omega	0.918/0.921	

EFA, Exploratory Factor Analysis (oblimin rotation, ML estimator); CFA, Confirmatory Factor Analysis (MLR estimator); AVE, Average Variance Extracted; CR, Composite Reliability. EFA and CFA were conducted on the same sample (*N* = 423) due to sample size constraints; results should therefore be interpreted with the caution noted in the Limitations section.

#### 2.2.3 Psychological wellbeing scale

Psychological wellbeing was evaluated using the scale developed by Diener et al. (2010) and adapted into Turkish by Telef (2013). This instrument measures core elements of positive human functioning, such as having supportive relationships, feeling competent, optimism, and leading a purposeful life. It consists of 8 items on a 7-point Likert-type scale (1 = strongly disagree, 7 = strongly agree) and operates as a unidimensional construct. CFA conducted in this study confirmed the single-factor structure with excellent fit indices (CFI = 0.913, TLI = 0.978, RMSEA = 0.035, SRMR = 0.050). The construct’s convergent validity was strongly supported (AVE = 0.531, CR = .900). Reliability analysis indicated high internal consistency, with a Cronbach’s alpha of 0.897 and a McDonald’s omega of 0.900 (Table 4).

**TABLE 4:** Exploratory and confirmatory factor analysis results for the future anxiety scale.

Items	EFA factor loading	CFA factor loading
Factor 1: Fear of the Future (α = 0.943, AVE = 0.563, CR = 0.943)		
1. I fear the possibility that my current problems will last for a long time	0.685	0.683
3. I am afraid of failing in the future	0.693	0.725
5. I am afraid that things will go wrong in my life	0.809	0.790
7. I fear not being able to overcome difficulties in the future	0.748	0.758
9. Even when everything is going well, I fear the possibility of a setback	0.765	0.762
11. Even when things are going well, I have the thought that something bad will happen	0.825	0.822
13. I am afraid of what the future will bring	0.802	0.825
14. I am anxious about not being able to provide good financial conditions for my family	0.747	0.745
15. The thought of my plans being left unfinished devastates me	0.738	0.746
16. The future looks blurry and uncertain to me	0.723	0.755
17. I am afraid that economic and political changes will threaten my future	0.805	0.767
18. I am afraid of the thought of making important decisions in the future	0.716	0.723
19. I am afraid that a great disaster will happen soon	0.658	0.633
Factor 2: Hopelessness about the Future (α = 0.849, AVE = 0.503, CR = 0.855)		
2. I think I will be happier in the future*	0.729	0.744
4. I hope I will be able to achieve the things I desire in the future*	0.859	0.837
6. My past experiences have prepared me well for the future*	0.648	0.595
8. I look to the future with hope and enthusiasm*	0.648	0.714
10. I have enough time to realize the things I really want to do*	0.500	0.510
12. I believe I will be able to achieve my goals in the future*	0.791	0.799
**Model fit statistics**	**EFA**	**CFA**
Eigenvalue	F1: 7.357/F2: 3.048	
Variance explained (%)	F1: %38.7/F2: %16.0 (Total: %54.8)	
KMO/Bartlett’s test	0.928/Chi-square = 5092.9, *p* < 0.001	
χ^2^/df	748.36/151	
CFI/TLI	0.881/0.865	
RMSEA [%90 CI]	0.097 [0.090–0.104]	
SRMR	0.059	
Inter-factor correlation (r)	0.369***	
Cronbach Alpha/McDonald Omega	0.926/0.943	

* Items with an asterisk are reverse-scored. EFA, Exploratory Factor Analysis (oblimin rotation, ML estimator); CFA, Confirmatory Factor Analysis (MLR estimator); AVE, Average Variance Extracted; CR, Composite Reliability. Inter-factor correlation: *r* = 0.369, ***p < 0.001. EFA and CFA were conducted on the same sample (*N* = 423) due to sample size constraints; results should therefore be interpreted with the caution noted in the Limitations section.

While the core SEM analyses were conducted on the full sample (*N* = 423), supplementary exploratory analyses (Cluster and CHAID) were performed utilizing listwise deletion for missing demographic data (e.g., age or sibling count), resulting in functional sub-samples of *n* = 338 and *n* = 359, respectively.

### 2.3 Statistical analysis

All analyses were conducted in R (R Core Team, 2020), with data management handled through the readxl (Wickham and Bryan, 2023) and dplyr (Wickham et al., 2023) packages. Descriptive statistics and exploratory factor analyses were carried out using the psych package (Revelle, 2024). To examine the structural validity of the measurement instruments, confirmatory factor analysis (CFA) was performed using the lavaan package (Rosseel, 2012) with a maximum likelihood robust (MLR) estimator. In line with recent recommendations, McDonald’s omega was reported alongside Cronbach’s alpha as a more comprehensive index of reliability (McDonald, 1999).

Distributional properties of continuous variables were evaluated through skewness and kurtosis coefficients. While scores on the Future Anxiety Scale and its subscales approximated normality, kurtosis values for the Psychological Wellbeing Scale and the Mental Toughness Inventory were somewhat elevated. Tabachnick and Fidell (2013) note that kurtosis values approaching or exceeding ± 3 warrant methodological attention; given this, the use of the MLR estimator and bootstrap confidence intervals in the SEM and mediation analyses was considered appropriate for obtaining more robust estimates. Normal distribution assumptions were evaluated against the criteria outlined by George and Mallery (2010) and Tabachnick and Fidell (2013). Linear associations among variables were examined using Pearson correlation analysis, and hierarchical regression analysis was conducted with variance inflation factors (VIF) checked to rule out multicollinearity (Field, 2018).

Mediation and moderation analyses were embedded within a structural equation modeling (SEM) framework. To estimate indirect effects, a non-parametric bootstrap procedure with 5,000 resamples was applied, yielding bias-corrected 95% bootstrap confidence intervals (95% BCa CI) (MacKinnon et al., 2004; Preacher and Hayes, 2008). The moderating role of gender was examined through multi-group SEM. Given that only the equivalence of the indirect effect across groups was tested rather than full sequential measurement invariance (configural, metric, scalar), this is reported as structural equivalence rather than measurement invariance, while the effect of income level was assessed via an interaction term derived from mean-centered variables. Specifically, the structural paths were freely estimated across gender groups in an unconstrained model, and the indirect effect (Future Anxiety → Mental Toughness → Psychological Wellbeing) was then compared between groups using a chi-square difference test on the constrained versus unconstrained models; a non-significant Δχ^2^ indicates that the strength of the indirect path does not differ meaningfully by gender, whereas the significant Δχ^2^ reported for individual structural paths reflects group differences in specific direct effects rather than in the overall mediation mechanism. As supplementary analyses, canonical correlation analysis was conducted using R’s base cancor function (Tabachnick and Fidell, 2013); K-means and hierarchical clustering (Ward.D2 linkage) were applied to identify student subprofiles (Kaufman and Rousseeuw, 1990); and a CHAID decision tree was fitted using the partykit package (Hothorn and Zeileis, 2015). Group difference tests relied on independent samples *t*-tests, one-way ANOVA, and Tukey HSD *post-hoc* comparisons. A significance threshold of *p* < 0.05 was applied throughout.

#### 2.3.1 The present study

The literature on future anxiety and psychological wellbeing has developed along two largely separate tracks. Öztekin (2025) demonstrated the association in a general undergraduate sample, but the explanatory mechanism there was optimism and pessimism—dispositional traits with no particular connection to any occupational domain. Koçeroğlu et al. (2024), meanwhile, worked directly with sport sciences students and isolated hopelessness as the most consequential facet of future anxiety, yet stopped short of asking what this means for wellbeing, or whether a resource specific to athletic training might explain it. Neither line of work, in other words, has asked whether mental toughness—a capacity built through competition and physical training rather than through general coping—does the explanatory work that optimism does elsewhere. That is the question this study was designed to answer. We test it in three steps. The first is whether mental toughness accounts for part of the association between future anxiety and wellbeing, rather than that association running directly, as it has been modeled in non-athletic samples. The second concerns which facet of future anxiety is doing the work: because hopelessness and fear of the future are conceptually distinct, collapsing them into a single score risks obscuring which one actually depresses mental toughness and wellbeing, so we examine them separately. The third is whether this mechanism holds uniformly across students or is conditioned by income and gender—not simply whether these variables predict the outcomes on their own, but whether the mediation pathway itself shifts across groups, which requires multi-group structural equation modeling rather than a simple moderated-regression approach.

Sport sciences students are not an arbitrary convenience sample for this purpose. Their transition into professional life carries the ordinary uncertainty of any graduating cohort, compounded by uncertainties specific to athletic careers—injury risk, competitive selection, and a limited window in which physical performance is marketable. It is this second layer that makes mental toughness a plausible mediator here in a way it would not be in a general student population, since mental toughness is cultivated specifically through athletic training and competition rather than through coursework or general life experience. On this basis, we hypothesized that future anxiety would negatively predict both psychological wellbeing (H1) and mental toughness (H2); that mental toughness, in turn, would positively predict wellbeing (H3) and would partially explain the future anxiety–wellbeing link (H4); and that this mechanism would not operate identically across all students, being shaped by income level (H5) and gender (H6). Given the cross-sectional design, these hypotheses were tested as structural associations consistent with the proposed mechanism, not as evidence of causal sequence.

### 2.4 Hypotheses

Drawing on prior research and theoretical frameworks concerning future anxiety, mental toughness, and psychological wellbeing, the following hypotheses were tested specifically within the population of sport sciences students:

*H1:* Future anxiety is directly and negatively associated with psychological wellbeing among sport sciences students.

*H2:* Future anxiety is negatively associated with the mental toughness of sport sciences students.

*H3*: Mental toughness is positively associated with psychological wellbeing among sport sciences students.

*H4*: Mental toughness mediates the relationship between future anxiety and psychological wellbeing within the sport sciences student population.

*H5*: Income level moderates the negative association between future anxiety and psychological wellbeing among sport sciences students, such that this detrimental effect weakens as their income level increases.

*H6*: Gender moderates the predictive pathways linking future anxiety, mental toughness, and psychological wellbeing within the sport sciences student sample.

### 2.5 Theoretical framework

Future anxiety is broadly defined as a persistent apprehension concerning possible adverse future events (Zaleski, 1996). For the specific population of sport sciences students, this anxiety is deeply intertwined with their unique educational and professional realities. These students do not merely face general academic stress; they confront a highly competitive sports industry, rigorous physical performance demands, and significant uncertainties regarding post-graduation athletic or coaching careers (Geylani and Çiriş Yıldız, 2022). Constant exposure to such career-specific unpredictability acts as a severe cognitive burden. When sport sciences students perceive their future in the sports sector as threatened, their capacity to maintain positive functioning and existential purpose—core components of psychological wellbeing—may be undermined (Ryff, 1989; Dalmış et al., 2025). Therefore, we hypothesized that future anxiety negatively predicts psychological wellbeing specifically among sport sciences students (H1).

Within the sport sciences context, mental toughness is a foundational psychological resource, cultivated through athletic training and competition (Gucciardi et al., 2015; Gerber et al., 2018). However, when sport sciences students experience intense future anxiety—particularly hopelessness regarding their careers in the sports domain—this vital resource is compromised. The cognitive load imposed by career-related ambiguities interferes with the self-regulation, attention, and goal-directed persistence that typically characterize an athlete’s mental toughness (Di Fronso et al., 2022). Extending this logic to the present sample, the chronic worry about unfulfilled athletic or professional expectations may be associated with lower psychological endurance, leading to a negative association between future anxiety and mental toughness (H2).

Mental toughness refers to the capacity to maintain focus, confidence, and persistence under demanding conditions—traits inherently essential for sport sciences students (Cowden, 2017). In this population, students with high mental toughness are better equipped to appraise the stressful transition from university to the competitive sports labor market as a manageable challenge rather than a debilitating threat. This adaptive coping mechanism directly supports their psychological wellbeing by preserving their sense of environmental mastery and purpose (Hu et al., 2015). Consequently, we anticipated a robust positive relationship between mental toughness and psychological wellbeing among sport sciences students (H3). Furthermore, integrating these dynamics into a unified model, we hypothesized that mental toughness serves as a psychological bridge: future anxiety is negatively associated with the psychological wellbeing of sport sciences students, and this association may be partially explained by lower levels of sport-specific mental toughness (H4).

Beyond these core psychological mechanisms, demographic factors such as socioeconomic status and gender significantly shape how sport sciences students experience future-oriented uncertainty. In the context of sports education, transitioning to professional athletic or coaching careers often involves financial instability and requires personal investment in specialized training, equipment, or certifications. Consequently, sport sciences students with higher socioeconomic resources may perceive this career ambiguity as less threatening, as their financial safety net buffers the psychological toll of future uncertainty (İleri, 2019; Kağıtçıbaşı, 1973; Tüzün and Çetinceli, 2022). Therefore, we hypothesized that income level moderates the negative association between future anxiety and psychological wellbeing among sport sciences students, such that higher income serves as a protective buffer (H5).

Furthermore, the sports industry and sport sciences education often present distinct structural challenges, performance expectations, and motivational climates for male and female athletes. Previous research indicates that psychological resources, self-efficacy, and wellbeing can vary significantly across gender groups (Pajares, 2003; Telef, 2013). Given these distinct competitive and psychological dynamics within the sports context, we explored whether gender acts as an additional moderating factor in the predictive pathways linking future anxiety, mental toughness, and psychological wellbeing among sport sciences students (H6).

## 3 Results

### 3.1 Descriptive statistics and correlations

Psychological wellbeing (*M* = 43.61, SD = 8.50) and mental toughness scores (*M* = 43.91, SD = 8.54) both fell above the theoretical midpoint of their respective scales, while future anxiety scores were slightly above the midpoint of that scale (*M* = 50.14, SD = 14.92) (Table 5).

**TABLE 5:** Descriptive statistics and normality indicators of the study variables.

Variable	N	Mean	SD	Min	Max	Skewness	Kurtosis
PWB total	423	43.61	8.50	8	56	–1.716	3.662
MTI total	423	43.91	8.54	8	56	–1.668	4.171
FAS total	423	50.14	14.92	19	91	–0.053	–0.301
FAS F1 fear	423	36.41	12.68	13	65	–0.041	–0.438
FAS F2 hopelessness	423	13.73	4.71	6	30	0.674	0.764
Gender	423	0.43	0.50	0	1	0.290	–1.920
Age group	359	1.43	0.50	1	2	0.263	–1.936
Income level	423	2.02	0.43	1	3	0.100	2.254
Number of siblings	388	2.57	0.85	1	4	–0.066	–0.619
Antidepressant use	423	0.06	0.24	0	1	3.556	10.670
Psychological treatment history	423	0.11	0.31	0	1	2.544	4.484

PWB, Psychological WellBeing Scale (8–56); MTI, Mental Toughness Inventory (8–56); FAS, Future Anxiety Scale (19–95). Skewness and kurtosis values within the range of ± 2 indicate that the assumption of normal distribution is met (George and Mallery, 2010).

Pearson correlations indicated that future anxiety was negatively and moderately associated with both psychological wellbeing (*r* = –0.378, *p* < 0.001) and mental toughness (*r* = –0.340, *p* < 0.001). In contrast, mental toughness and psychological wellbeing were strongly and positively related (*r* = 0.770, *p* < 0.001) (Table 6).

**TABLE 6:** Pearson correlation matrix of the study variables.

Variable	PWB	MTI	FAS	FAS F1	FAS F2	Gender	Age	Income	Siblings	Antidep.	Treatment
1. PWB	(0.897)	(0.918)	(0.926)	(0.943)	(0.849)	(–)	(–)	(–)	(–)	(–)	(–)
2. MTI	0.770***										
3. FAS	–0.378***	–0.340***									
4. FAS F1	–0.226***	-0.187***	0.954***								
5. FAS F2	–0.589***	–0.571***	0.596***	0.329***							
6. Gender	–0.126**	–0.036	–0.003	–0.030	0.070						
7. Age	0.052	0.058	–0.162**	–0.146**	–0.111*	–0.152**					
8. Income	0.285***	0.217***	–0.104*	–0.070	–0.140**	0.006	0.047				
9. Siblings	0.163**	0.120*	–0.049	–0.064	0.015	–0.010	0.032	0.037			
10. Antidep.	–0.252***	–0.140**	0.158**	0.136**	0.134**	0.146**	–0.032	-0.123*	–0.059		
11. Treatment	–0.192***	–0.019	0.146**	0.143**	0.077	0.043	–0.124*	-0.191***	–0.191***	0.474***	

Values in parentheses on the diagonal represent Cronbach’s alpha reliability coefficients. PWB, Psychological WellBeing; MTI, Mental Toughness Inventory; FAS, Future Anxiety Scale; FAS F1, Fear of the Future; FAS F2, Hopelessness about the Future; Antidep., Antidepressant Use; Treatment, Psychological Treatment History. *p < 0.05. **p < 0.01. ***p < 0.001.

#### 3.1.1 Direct effects and the mediating role of mental toughness (testing H1, H2, H3, and H4)

Structural equation modeling (SEM) was used to examine the mechanisms linking future anxiety to psychological wellbeing. Future anxiety exerted a direct, negative, and statistically significant effect on psychological wellbeing (β = –0.131, *p* < 0.001), supporting H1. Future anxiety was also negatively associated with mental toughness (pathway a: β = –0.340, *p* < 0.001), consistent with H2. Mental toughness, in turn, was positively and strongly associated with psychological wellbeing (pathway b: β = 0.725, *p* < 0.001), supporting H3.

The mediation analysis confirmed that future anxiety was indirectly associated with psychological wellbeing through mental toughness [*B* = –0.141, β = –0.247, 95% BCa CI (–0.186, –0.099), *p* < 0.001], with the confidence interval excluding zero indicating a statistically significant indirect effect. Importantly, the direct effect of future anxiety on wellbeing remained significant after accounting for the mediator (*B* = –0.075, β = –0.131, *p* < 0.001), establishing mental toughness as a partial rather than full mediator. H4 was therefore supported (Table 7). Examining the subscale-level results, the Hopelessness about the Future subscale showed the strongest indirect association with psychological wellbeing through mental toughness [*B* = –0.663, β = –0.368, 95% BCa CI (–0.855, –0.492), *p* < 0.001] (Tables 8, 9).

**TABLE 7:** Structural equation modeling results for the mediating role of mental toughness in the relationship between future anxiety and psychological wellbeing (model 1).

Pathway	*B*	SE	*z*	*p*	β	%95 CI lower	%95 CI higher
FAS → MTI (pathway a)	–0.195	0.030	-6.542	0.000	–0.340	–0.250	–0.135
MTI → PWB (pathway b)	0.722	0.048	14.884	0.000	0.725	0.619	0.811
FAS → PWB (direct effect, c’)	–0.075	0.016	-4.687	0.000	–0.131	–0.107	–0.044
Indirect effect (a × b)	–0.141	0.022	–6.256	0.000	–0.247	–0.186	–0.099
Total effect (c)	–0.215	0.026	–8.182	0.000	–0.378	–0.267	–0.164
Model fit: CFI = 1.000 | TLI = 1.000 | RMSEA = 0.000 | SRMR = 0.000 | Bootstrap = 5,000 | %95 BCa CI.							

B, unstandardized path coefficient; β, standardized path coefficient; SE, standard error; CI, confidence interval; FAS, Future Anxiety Scale; MTI, Mental Toughness Inventory; PWB, Psychological WellBeing. Bootstrap: 5,000 resamples. Bias-corrected (BCa) 95% confidence intervals are reported. *p < 0.05. **p < 0.01. ***p < 0.001.

**TABLE 8:** Structural equation modeling results for the mediating role of mental toughness in the relationship between fear of the future and psychological wellbeing (model 2).

Pathway	*B*	SE	*z*	*p*	β	%95 CI lower	%95 CI higher
FAS F1 → MTI (pathway a)	–0.126	0.041	–3.071	0.002	–0.187	–0.205	–0.042
MTI → PWB (pathway b)	0.751	0.047	16.134	0.000	0.754	0.651	0.835
FAS F1 → PWB (direct effect, c’)	–0.056	0.020	–2.883	0.004	–0.084	-0.096	-0.020
Indirect effect (a × b)	–0.095	0.030	–3.164	0.002	–0.141	–0.151	–0.033
**Total effect (c)**	–0.151	0.039	–3.881	0.000	–0.226	–0.226	–0.073
Model fit: CFI = 1.000 | TLI = 1.000 | RMSEA = 0.000 | SRMR = 0.000 | Bootstrap = 5,000 | %95 BCa CI							

B, unstandardized path coefficient; β, standardized path coefficient; SE, standard error; CI, confidence interval; FAS F1, Fear of the Future; MTI, Mental Toughness Inventory; PWB, Psychological WellBeing. Bootstrap: 5,000 resamples. Bias-corrected (BCa) 95% confidence intervals are reported. *p < 0.05. **p < 0.01. ***p < 0.001.

**TABLE 9:** Structural equation modeling results for the mediating role of mental toughness in the relationship between future hopelessness and psychological wellbeing (model 3).

Pathway	*B*	SE	*z*	*p*	β	%95 CI lower	%95 CI higher
FAS F2 → MTI (pathway a)	–1.035	0.111	–9.302	0.000	–0.571	–1.247	–0.812
MTI → PWB (pathway b)	0.641	0.050	12.732	0.000	0.644	0.541	0.737
FAS F2 → PWB (direct effect, c’)	–0.398	0.069	–5.804	0.000	–0.221	–0.527	–0.259
Indirect effect (a × b)	–0.663	0.093	–7.105	0.000	–0.368	–0.855	-0.492
Total effect (c)	–1.061	0.099	–10.708	0.000	–0.589	–1.249	–0.870
Model fit: CFI = 1.000 | TLI = 1.000 | RMSEA = 0.000 | SRMR = 0.000 | Bootstrap = 5,000 | %95 BCa CI							

B, unstandardized path coefficient; β, standardized path coefficient; SE, standard error; CI, confidence interval; FAS F2, Hopelessness about the Future; MTI, Mental Toughness Inventory; PWB, Psychological WellBeing. Bootstrap: 5,000 resamples. Bias-corrected (BCa) 95% confidence intervals are reported. *p < 0.05. **p < 0.01. ***p < 0.001.

#### 3.1.2 Moderating roles of income level and gender (testing H5 and H6)

The moderation analysis indicated that income level significantly moderated the association between future anxiety and psychological wellbeing (interaction term: β = 0.190, *p* < 0.001). The positive direction of the interaction term suggests that as income level increases, the negative impact of future anxiety on wellbeing diminishes; in other words, higher socioeconomic resources appear to buffer students against the psychological costs of future-oriented uncertainty. Given the pronounced imbalance across income categories (see Limitations), this finding should be regarded as preliminary support for H5 rather than a confirmatory result (Table 10).

**TABLE 10:** Moderating effect of income level on the relationship between future anxiety and psychological wellbeing.

Predictor	*B*	SE	*z*	*p*	β	%95 CI lower	%95 CI higher
FAS (direct effect, c)	–0.185	0.021	–8.791	0.000	–0.325	–0.225	–0.143
Income level (m)	4.390	0.836	5.249	0.000	0.225	2.791	6.114
FAS × income level	0.256	0.065	3.947	0.000	0.190	0.125	0.379
*R*^2^ = 0.239 | CFI = 1.000 | RMSEA = 0.000 | Bootstrap: 5,000 resamples							

Variables were mean-centered. B, unstandardized path coefficient; β, standardized path coefficient; SE, standard error; CI, confidence interval; FAS, Future Anxiety Scale. Bootstrap: 5,000 resamples. Bias-corrected (BCa) 95% confidence intervals are reported. *p < 0.05. **p < 0.01. ***p < 0.001.

Multi-group SEM was used to examine the moderating role of gender. The results indicated that gender introduced structural differences in certain path coefficients (Δχ^2^ = 18.924, *p* < 0.001): specifically, the positive effect of mental toughness on psychological wellbeing was stronger in males (β = 0.780) than in females (β = 0.653). However, a conditional indirect effect analysis testing the structural equivalence of the full mediation chain (Future Anxiety → Mental Toughness → Psychological WellBeing) found that the indirect effect did not differ meaningfully between gender groups (B = -0.135, β = -0.213; Δχ^2^ = 0.000, n.s.). H6 was therefore only partially supported (Tables 11, 12).

**TABLE 11:** Multi-group structural equation modeling results by gender.

Pathway	Male (*n* = 181)			Female (*n* = 242)		
	***B***	**β**	***p***	***B***	**β**	***p***
FAS → MTI (pathway a)	–0.218	–0.324	0.000	–0.181	–0.360	0.000
MTI → PWB (pathway b)	0.833	0.780	0.000	0.583	0.653	0.000
FAS → PWB (pathway c’)	–0.116	–0.161	0.000	–0.061	–0.136	0.003
Indirect effect (a × b)	–0.182	–0.252	0.000	–0.105	–0.235	0.000
Konfigural: CFI = 1.000, RMSEA = 0.000 | Metrik: CFI = 0.951, RMSEA = 0.185 | LRT: Δχ^2^ = 18.924, *p* < 0.001						

Configural model: paths are freely estimated across groups. Metric model: regression paths are constrained to be equal across groups. LRT, Likelihood Ratio Test; FAS, Future Anxiety Scale; MTI, Mental Toughness Inventory; PWB, Psychological WellBeing. *p < 0.05. **p < 0.01. ***p < 0.001.

**TABLE 12:** Conditional mediation analysis results across gender groups.

Pathway	Male (*n* = 181)			Female (*n* = 242)		
	***B***	**β**	***p***	***B***	**β**	***p***
FAS → MTI (pathway a)	–0.192	–0.288	0.000	–0.192	–0.378	0.000
MTI → PWB (pathway b)	0.705	0.738	0.000	0.705	0.706	0.000
FAS → PWB (pathway c’)	–0.073	–0.116	0.000	–0.073	–0.145	0.000
Indirect effect (a × b)	–0.135	–0.213	0.000	–0.135	–0.213	0.000
Δχ^2^ = 0.000 (*p* = n.s.) —Indirect effect demonstrates structural equivalence across gender groups. | Bootstrap: 5,000						

Bootstrap: 5,000 resamples. n.s., not statistically significant. FAS, Future Anxiety Scale; MTI, Mental Toughness Inventory; PWB, Psychological WellBeing. *p < 0.05. **p < 0.01. ***p < 0.001.

### 3.2 Canonical correlation, cluster, and CHAID decision tree analyses

Canonical correlation analysis identified two statistically significant canonical roots (both *p* < 0.001). The first root (Rc = 0.697, Rc^2^ = 0.485) was dominated by psychological wellbeing on the Y-side (structure coefficient = -0.999), with antidepressant use (0.803) and income level (-0.590) emerging as the strongest contributors on the X-side. This pattern suggests that students reporting antidepressant use and lower income levels tend to show a less favorable psychological wellbeing profile. Cluster analysis—conducted using both K-means and hierarchical (Ward.D2) methods on the *n* = 338 participants with complete data across all clustering variables—yielded two distinct profiles. Cluster 1, comprising 72.5% of that subsample, reflected an adaptive or resilient profile characterized by high psychological wellbeing, high mental toughness, and low future anxiety. Cluster 2 (27.5%) presented the opposite pattern -lower wellbeing, lower mental toughness, and elevated future anxiety- and was characterized as a vulnerable profile (Table 13). The CHAID decision tree analysis further showed that mental toughness was the primary splitting variable in the classification of psychological wellbeing outcomes. This should be read as an exploratory finding rather than a confirmatory one. Within the lower-to-moderate mental toughness group, age emerged as the secondary differentiating variable: notably, 86.1% of students aged 22–24 with low mental toughness fell into the low wellbeing category (Table 14).

**TABLE 13:** K-means and hierarchical cluster analysis results.

Variable	Cluster 1 (*n* = 245, %72.5)	Cluster 2 (*n* = 93, %27.5)
PWB (M ± SD)	46.28 ± 7.12	35.56 ± 8.34
MTI (M ± SD)	46.25 ± 7.42	36.40 ± 8.87
FAS total (M ± SD)	46.28 ± 14.24	60.56 ± 13.51
FAS F1 fear (M ± SD)	33.83 ± 12.44	43.71 ± 12.08
FAS F2 hopelessness (M ± SD)	12.45 ± 4.39	16.85 ± 4.60
Income level (M)	2.06	1.90
Antidepresan (%)	4.5%	12.9%
Psychological treatment (%)	8.6%	16.1%
Cluster profile	High wellbeing, low anxiety	Low wellbeing, high anxiety
Silhouette coefficient	0.247	
Hierarchical fit (Ward.D2)	76.8% overlap with K-means	

The optimal number of clusters was determined as *k* = 2 using the silhouette (0.247) and elbow methods. Profile definitions were based on standardized cluster centers.

**TABLE 14:** CHAID decision tree node distributions.

Node	Rule	n	Low %%	Medium %	High %	Dominant
Root	Total sample	359	35.4%	43.5%	21.2%	Medium
2	MTI = Low/medium; age = 18–21	61	73.8%	26.2%	0.0%	Low
3	MTI = Low/medium; age = 22–24	36	86.1%	13.9%	0.0%	Low
4	MTI = high; FAS = low	90	17.8%	64.4%	17.8%	Medium
5	MTI = high; FAS = medium/high	172	29.7%	45.9%	24.4%	Medium
Overall accuracy: 59.33% | Internal nodes:: 3 | Terminal nodes: 4 | α = 0.05 | Min. node size = 20						

The dependent variable PWB was divided into three categories (low/medium/high). α = 0.05, minimum node size = 20, maximum depth = 4.

These additional analyses (Canonical correlation, K-means clustering, and CHAID decision tree) were conducted solely as supplementary, exploratory procedures rather than confirmatory tests of the core theoretical model. They aim to provide a broader profiling of the sample, but their results should be interpreted cautiously within the cross-sectional boundaries of this study.

#### 3.2.1 Group difference analyses (independent samples *t*-tests and ANOVA)

Female students reported significantly higher psychological wellbeing than male students (*p* = 0.011). Income level was associated with meaningful differences in both psychological wellbeing [*F*(2, 420) = 8.76, *p* < 0.001] and mental toughness [*F*(2, 420) = 5.29, *p* = 0.005], with Tukey HSD *post-hoc* comparisons indicating that the high-income group scored significantly higher than both the low- and middle-income groups on each measure (Table 15).

**TABLE 15:** Comparison of scale scores across demographic characteristics (M ± SD).

Independent variable/groups	n	PWB	MTI	FAS total	FAS F1 fear	FAS F2 Hop.
Gender (independent t-test)						
Male	181	42.41 ± 8.89	43.74 ± 8.85	50.18 ± 15.65	36.44 ± 13.27	13.74 ± 4.87
Female	242	44.46 ± 8.11	44.03 ± 8.31	50.11 ± 14.40	36.39 ± 12.26	13.72 ± 4.60
*t*(421)/p		-2.54/0.011*	–0.35/0.727	0.05/0.959	0.04/0.968	0.04/0.969
Cohen d		0.25	0.04	0.01	0.00	0.00
Antidepressant use (Indep. t-test)						
Yes	27	38.22 ± 9.87	40.26 ± 10.45	54.74 ± 17.23	39.56 ± 14.22	15.19 ± 5.47
No	396	43.88 ± 8.33	44.10 ± 8.38	49.79 ± 14.72	36.16 ± 12.57	13.63 ± 4.68
*t*(421)/p		-3.27/0.001**	–2.09/0.037*	1.68/0.094	1.38/0.170	1.73/0.085
Cohen d		0.61	0.40	0.30	0.26	0.33
Psych. treatment history (t-test)						
Yes	45	40.02 ± 9.65	43.73 ± 9.12	52.89 ± 15.78	38.44 ± 13.45	14.44 ± 4.98
No	378	43.87 ± 8.35	43.93 ± 8.51	49.75 ± 14.82	36.16 ± 12.58	13.60 ± 4.69
*t*(421)/p		-2.64/0.009**	–0.14/0.890	1.25/0.213	1.09/0.277	1.07/0.286
Cohen d		0.43	0.02	0.21	0.18	0.18
Age group (one-way ANOVA)						
18–21 (a)	203	43.30 ± 8.45	43.64 ± 8.45	51.61 ± 14.72	37.42 ± 12.56	14.19 ± 4.78
22–24 (b)	156	44.08 ± 8.60	44.44 ± 8.76	49.04 ± 14.95	35.47 ± 12.76	13.57 ± 4.58
25–27 (c)	21	45.14 ± 7.92	46.19 ± 7.34	46.24 ± 14.12	33.57 ± 11.84	12.67 ± 4.23
28 + (d)	43	42.53 ± 9.11	41.93 ± 8.89	52.07 ± 16.44	38.12 ± 13.77	13.95 ± 5.01
*F*(df)/p		2.14(3) /0.096	1.53(3)/0.207	3.26(3)/0.021*	2.26(3)/0.081	1.36(3)/0.255
Income level (one-way ANOVA)						
Low (a)	36	39.14 ± 9.18	40.28 ± 9.34	53.58 ± 16.12	38.94 ± 13.87	14.64 ± 5.25
Middle (b)	343	43.65 ± 8.39	43.94 ± 8.47	50.21 ± 14.87	36.49 ± 12.66	13.72 ± 4.69
High (c)	44	47.45 ± 7.62	47.07 ± 7.89	47.16 ± 14.02	34.27 ± 11.98	12.89 ± 4.47
*F*(df)/p		8.76(2)/0.000***	5.29(2)/0.005**	1.89(2)/0.153	1.97(2)/0.141	1.52(2)/0.220
*Post-hoc*		a < c; b < c	a < c; b < c	-	-	-
Region (one-way ANOVA)						
Black sea (a)	331	43.51 ± 8.48	43.73 ± 8.55	51.02 ± 14.86	37.07 ± 12.70	13.95 ± 4.72
Marmara (b)	31	42.16 ± 8.74	39.45 ± 8.43	58.39 ± 14.78	43.38 ± 13.38	15.00 ± 5.18
Mediterranean (c)	15	44.27 ± 8.13	45.80 ± 8.24	48.67 ± 13.44	34.87 ± 11.22	13.80 ± 4.53
Other (d)	46	44.13 ± 8.71	45.41 ± 8.31	46.22 ± 14.62	33.96 ± 12.03	12.26 ± 4.48
*F*(df)/p		0.49(6)/0.816	2.97(6)/0.008**	2.81(6)/0.011*	3.04(6)/0.007**	1.20(6)/0.306
Siblings (one-way ANOVA)						
1 (a)	40	40.45 ± 9.37	46.03 ± 8.04	52.40 ± 16.12	39.65 ± 14.29	12.75 ± 4.19
2 (b)	139	43.41 ± 8.47	44.53 ± 8.49	49.52 ± 14.75	35.95 ± 12.76	13.57 ± 4.65
3 (c)	157	44.08 ± 8.28	43.81 ± 8.72	50.23 ± 14.54	36.43 ± 12.38	13.80 ± 4.78
4+ (d)	52	46.28 ± 8.05	43.68 ± 9.03	50.88 ± 15.56	36.64 ± 13.27	14.24 ± 4.84
*F*(df)/p		3.97(3)/0.008**	2.24(3)/0.084	2.35(3)/0.072	2.82(3)/0.039*	0.28(3)/0.843
*Post-hoc*		a < d	-	-	a < c	-

M ± SD = Mean ± Standard Deviation. Cohen’s d was calculated for *t*-tests. *Post-hoc* comparisons were conducted using Tukey HSD. *p < 0.05. **p < 0.01. **p < 0.001.

## 4 Discussion

This study examined the direct and indirect associations between future anxiety and psychological wellbeing among sport sciences students, with mental toughness tested as a mediator and income level and gender examined as moderators. Overall, the findings indicate that future anxiety is negatively associated with psychological wellbeing and that this relationship is partially explained by mental toughness. The study also shows that income level serves as a buffer against the negative association between future anxiety and wellbeing, whereas gender influences specific path coefficients without changing the overall mediation mechanism. These findings contribute to the literature by examining future anxiety, mental toughness, and psychological wellbeing within an integrated mediation-moderation model in a sport sciences student sample.

### 4.1 The relationship between future anxiety and psychological wellbeing

One of the main findings of this study was the negative association between future anxiety and psychological wellbeing. In the simple regression model, future anxiety was a moderate negative predictor of psychological wellbeing (β = -0.378, *p* < 0.001). When mental toughness was included in the SEM model, the direct effect decreased but remained significant (β = -0.131, *p* < 0.001), indicating partial mediation. This aligns with prior evidence indicating that stress and anxiety deteriorate psychological health outcomes (Gerber et al., 2018; Hu et al., 2015). It is also in line with theoretical accounts of future anxiety, which describe future-oriented uncertainty and neurotic anxiety as factors that may impair psychological functioning (Zaleski, 1996; Geylani and Çiriş Yıldız, 2022). Among university students, concerns about unemployment, career uncertainty, and future life goals may contribute to lower psychological wellbeing (Kula and Saraç, 2016; Aygün, 2014). However, because the confirmatory factor analysis fit indices for the Future Anxiety Scale in the present sample were below or close to conventional thresholds (CFI = 0.881, RMSEA = 0.097), findings based on this scale should be interpreted cautiously.

Dalmış et al. (2025) reported that psychological resilience negatively predicted future anxiety and that subjective wellbeing mediated this relationship among university students. Although the direction of mediation differs from the present study, both findings indicate that anxiety, resilience-related constructs, and wellbeing are interrelated. This suggests that the relationship between future anxiety and wellbeing may be better understood through models that include psychological resources rather than through direct associations alone.

Öztekin (2025), in a sample of 1,024 undergraduate students, found that future anxiety was negatively associated with wellbeing and that optimism and pessimism mediated this relationship. The present finding that the Hopelessness about the Future subscale was strongly associated with psychological wellbeing (β = –0.589) strongly supports this result, as both pessimism and hopelessness reflect negative cognitive appraisals of future outcomes. In the present study, hopelessness was a stronger predictor of psychological wellbeing than fear of the future (β = –0.589 vs. β = –0.226), suggesting that the loss of positive future expectations may be particularly relevant for students’ wellbeing. A similar pattern was observed by Koçeroğlu et al. (2024), who investigated hopelessness and future anxiety among sport sciences students. Similarly, Taşğın et al. (2017) reported significant associations between unemployment anxiety and psychological resilience among university students. When viewed as a whole, this body of literature indicates that employment-related uncertainty and negative future expectations may be linked not only to higher future anxiety but also to lower psychological resources and wellbeing.

### 4.2 The effect of future anxiety on mental toughness

Future anxiety was negatively associated with mental toughness. This suggests that students who report higher levels of future-oriented anxiety may have more difficulty maintaining focus, confidence, and goal-directed persistence under pressure. These results parallel prior research demonstrating that perceived stress is associated with burnout and depressive symptoms among young athletes, while mental toughness may have a protective role under high-stress conditions (Gerber et al., 2018). Di Fronso et al. (2022) also emphasized the relevance of perceived stress and interoceptive awareness in sport contexts, suggesting that stress may influence how athletes monitor and respond to internal bodily and emotional signals. In the present study, future anxiety may represent a sustained psychological load that is negatively related to the mental toughness resources required for preparation, performance, and recovery. Therefore, the findings support the view that reducing future-oriented anxiety and strengthening mental toughness may both be relevant for protecting psychological wellbeing among sport sciences students.

### 4.3 The effect of mental toughness on psychological wellbeing

Mental toughness emerged as one of the strongest correlates of psychological wellbeing in this study. The bivariate association was strong (*r* = 0.770, *p* < 0.001), and the mediation model also showed a strong positive path from mental toughness to psychological wellbeing (β = 0.725, *p* < 0.001). Consistent with the present findings, Yılmaz et al. (2026) demonstrated that mental fatigue awareness mediated the relationship between perceived stress and psychological wellbeing among combat athletes. Although the mediating constructs differ, both studies emphasize that psychological processes play an important role in explaining the relationship between adverse psychological conditions and psychological wellbeing in athletic populations. These findings indicate that mental toughness is not only relevant for performance-related outcomes but is also closely associated with general psychological functioning. Such a strong relationship perfectly mirrors the core conceptualization of mental toughness as the capacity to maintain confidence, persistence, and effective functioning under demanding conditions (Gucciardi et al., 2015; Etlioğlu and Yılmaz, 2025). Previous studies also suggest that mental toughness and related resilience constructs are associated with more effective coping and better mental health outcomes under stress (Cowden, 2016; Cowden, 2017; Hu et al., 2015; Sahin, 2026). One possible explanation is that mentally tough individuals may be more likely to interpret stressful situations as manageable challenges rather than uncontrollable threats, which may support psychological wellbeing dimensions such as environmental mastery and purpose in life (Ryff, 1989). In addition, intervention-based studies have shown that structured combat sport training can improve quality of life and other positive psychological outcomes, including self-control, in healthy adults (Şahin et al., 2025). In the present sport sciences sample, exposure to performance demands, training discipline, and competitive experiences may also contribute to the development or maintenance of mental toughness. This interpretation is further supported by the relatively high mean mental toughness score observed in the sample (*M* = 43.91), although this should be tested directly in future studies including sport-specific variables. Erhan et al. (2021) similarly showed that psychological resilience mediated the relationship between career decidedness and psychological wellbeing, supporting the broader view that internal psychological resources may buffer the effects of adverse conditions on wellbeing.

### 4.4 The mediating role of mental toughness

The mediation analysis revealed that mental toughness partially explained the relationship between future anxiety and psychological wellbeing [*B* = –0.141, β = –0.247, 95% BCa CI (–0.186, –0.099), *p* < 0.001]. This finding indicates that future anxiety is associated with psychological wellbeing both directly and indirectly through mental toughness. In other words, students with higher future anxiety may report lower psychological wellbeing partly because they also report lower mental toughness. Our results show similarity with the studies highlighting that stress and anxiety compromise psychological health outcomes, while mental toughness and related resilience resources may play a protective role under demanding conditions (Gerber et al., 2018; Dalmış et al., 2025).

Benjamin and John (2021) reported that mental toughness functioned as both a moderator and a mediator in the relationship between perceived stress and anxiety, with stronger effects among athletes than non-athletes. Our data corroborate this dynamic, especially given that our sample consisted entirely of sport sciences students. It is possible that repeated exposure to performance demands, training discipline, and competitive contexts strengthens the relevance of mental toughness as a psychological resource in this group. However, this interpretation should be tested directly in future studies using sport-specific variables such as training history, competitive level, and physical activity intensity.

The subscale findings further showed that Hopelessness about the Future produced the strongest indirect effect in the mediation model (β = –0.368). This underscores the important role of negative future expectations in explaining the lower levels of both mental toughness and psychological wellbeing. Loss of positive future expectations may weaken students’ confidence in their goals and their perceived capacity to cope with future demands, thereby contributing to lower wellbeing. The prominent role of hopelessness directly supports theoretical models that position it as a critical risk factor in resilience and mental health research.

### 4.5 Moderating variables: income level and gender

The moderation findings tentatively suggest that income level buffers the relationship between future anxiety and psychological wellbeing (β = 0.190, *p* < 0.001), although this result warrants cautious interpretation given the imbalanced group sizes. This suggests that the negative association between future anxiety and wellbeing was weaker among students with higher income levels. It is highly probable that socioeconomic resources mitigate the psychological impact of future-oriented uncertainty by expanding perceived access to opportunities (Kağıtçıbaşı, 1973; İleri, 2019). Conversely, financial constraints may intensify the effects of future anxiety by limiting perceived control over post-graduation outcomes. These results lend support to Tüzün and Çetinceli (2022), who previously noted the protective role of socioeconomic factors against unemployment anxiety, life satisfaction, and psychological wellbeing among university students.

Gender-related analyses showed a more complex pattern. Female students reported higher psychological wellbeing than male students (*d* = 0.25), and multi-group SEM indicated that some path coefficients differed by gender (Δχ^2^ = 18.924, *p* < 0.001). In particular, the association between mental toughness and psychological wellbeing was stronger among male students (β = 0.780) than among female students (β = 0.653). However, the overall indirect effect of future anxiety on psychological wellbeing through mental toughness did not differ meaningfully between gender groups (Δχ^2^ = 0.000, n.s.). Therefore, gender appears to influence the magnitude of specific associations in the model, but not the general mediation mechanism. This pattern aligns with previous literature suggesting that self-efficacy, motivation, and psychological wellbeing may vary across gender groups (Pajares, 2003; Telef, 2013). Hence, the present findings should be interpreted as partial support for the moderating role of gender.

The canonical correlation analysis provided an exploratory view of the multivariate pattern among psychological, clinical, and socioeconomic variables. In the first canonical root, antidepressant use and income level emerged as the strongest contributors on the predictor side, whereas psychological wellbeing was the dominant variable on the outcome side. This pattern suggests that lower income level and antidepressant use were associated with a less favorable psychological wellbeing profile. These findings support the importance of considering socioeconomic and psychological support-related variables alongside individual psychological resources. The second canonical root, in which psychological treatment history and gender contributed more strongly, should be interpreted cautiously because canonical correlation analysis was used as an exploratory supplementary method rather than as a confirmatory hypothesis test.

### 4.6 Profiles: cluster and CHAID findings

The cluster analysis identified two student profiles. The first profile, representing 72.5% of the clustering subsample, was characterized by higher psychological wellbeing, higher mental toughness, and lower future anxiety. The second profile, representing 27.5% of the subsample, showed lower psychological wellbeing, lower mental toughness, and higher future anxiety. This second profile was also associated with higher rates of antidepressant use and psychological treatment history. These findings suggest that the vulnerable profile may reflect a subgroup of students with greater psychological support needs. Similar observations were made by Aksu and Kuas (2024), who reported that lower psychological resilience was associated with higher career anxiety among students.

The CHAID decision tree analysis further indicated that mental toughness was the primary variable differentiating psychological wellbeing outcomes. Among students with low or moderate mental toughness, age emerged as a secondary differentiating variable; 86.1% of students aged 22–24 in this subgroup were classified in the low wellbeing category. This finding suggests that the transition period associated with graduation, employment search, internships, and career decision-making may be particularly important for students with lower mental toughness. This reflects the findings of Koçeroğlu et al. (2024), who reported increased anxiety among sport sciences students approaching graduation. Because the CHAID analysis was exploratory, these findings should be interpreted as preliminary and should be tested in future studies.

### 4.7 Originality

The sport sciences context represents an important feature of the present study. Students in this field are educated within a setting that includes physical activity, performance expectations, competitive experiences, and training discipline. These contextual features may be relevant to the development and use of mental toughness as a psychological resource. The above-average psychological wellbeing scores observed in the present sample (*M* = 43.61) corroborate the work of Tunç (2020), who reported that sport sciences students show psychological wellbeing profiles comparable to those of general university student samples. However, because the present study did not include students from other faculties, no direct conclusion can be drawn regarding whether sport sciences students differ systematically from other student groups. Future comparative studies are needed to determine whether the observed patterns are specific to sport sciences education or reflect broader university student dynamics.

At the same time, the findings indicate that sport sciences students are not exempt from future-oriented concerns. Employment uncertainty, competitive labor conditions, and career-related ambiguity in the field may contribute to higher future anxiety and lower psychological wellbeing. Therefore, the present study contributes to the literature by showing that future anxiety, mental toughness, and psychological wellbeing are meaningfully interconnected within a sport sciences student sample.

## 5 Limitations and directions for future research

The current study has several methodological constraints. Primarily, the cross-sectional design and the reliance on single-time-point self-report measures preclude any definitive causal inferences and introduce the risk of common method variance. No procedural or statistical remedies (e.g., Harman’s single-factor test, marker-variable technique) were applied to assess or control for common method bias in this study, and this should be considered when interpreting the strength of the reported associations. Although the proposed model is theoretically grounded, longitudinal studies are needed to clarify the temporal order of these associations. Second, the measurement model for the Future Anxiety Scale yielded marginal fit indices (CFI = 0.881, RMSEA = 0.097), and all associated findings should be interpreted with caution. Third, due to sample size constraints (*N* = 423), both Exploratory Factor Analysis (EFA) and Confirmatory Factor Analysis (CFA) were conducted on the same dataset. Future studies should employ larger samples to allow for split-half cross-validation. Fourth, the income level variable was highly imbalanced (with only 36 low-income and 44 high-income participants compared to 343 middle-income). Consequently, treating income as a continuous moderator represents a limitation, and its moderating effect should be interpreted merely as exploratory. Finally, the omission of sport-specific variables—such as competitive level, physical activity intensity, and specific athletic training history—represents a major limitation. Including these specific athletic variables in future research may help clarify which aspects of sport sciences education most strongly relate to psychological wellbeing.

## 6 Conclusion

The present study provides evidence that future anxiety is negatively associated with psychological wellbeing among sport sciences students and that mental toughness partially explains this relationship. Students reporting higher future anxiety also tended to report lower mental toughness, which in turn was strongly associated with lower psychological wellbeing. Income level appeared to moderate the relationship between future anxiety and wellbeing, tentatively suggesting that socioeconomic resources may reduce the psychological impact of future-oriented uncertainty; however, this result should be confirmed in future studies with more balanced income groups. Gender differences were observed in some structural paths, particularly in the association between mental toughness and psychological wellbeing; however, the indirect effect did not differ meaningfully between male and female students. These findings suggest that mental toughness is an important psychological resource in sport sciences students and may be a useful target for preventive support programs, especially for students facing economic disadvantage or transition-related career uncertainty.

## Data Availability

The raw data supporting the conclusions of this article will be made available by the authors, without undue reservation.
